# Real frontier in clinical applications of regenerative medicine

**DOI:** 10.1186/s41232-020-00112-z

**Published:** 2020-03-26

**Authors:** Kazuo Tsubota

**Affiliations:** Department of Ophthalmology, Keio University School of Medicine, Tokyo, Japan

In this thematic series, Real Frontier in Clinical Applications of Regenerative Medicine in *Inflammation and Regeneration* four leading research teams showcase their review articles as the forefront of regenerative medicine in clinical applications in Japan. We are very proud of the progress made over the last several years.

As we know, there are three kinds of approaches to regenerative medicine. The first is cell therapy, the second is protein, and the third applies artificial materials. One such technique that was developed in Japan using a local injection of a gelatin hydrogel impregnated with the protein fibroblast growth factor-2 is gaining in popularity, and the clinical application of this method is expanding. Dr. Kuroda from Kyoto University reviewed the clinical application of injectable growth factor for bone regeneration, and his review describes the progress of injectable growth factor, not implanting regenerative tissue, but supporting the regeneration inside the body.

From Keio University, Drs. Hatou and Shimmura reviewed corneal endothelial cell derivation methods from embryonic stem (ES) and induced pluripotent (iPS) cells. Dr. Hatou has developed a method of regeneration from iPS cells to make endothelial cells, and it will be ready for the clinical application soon. They have taken their research seriously and formed a start-up company, Cellusion, Inc. The method can save sight for patients around the world who are suffering from corneal endothelial dysfunction.

The research led by Drs. Tsuchiya and Terai from Niigata University report on mesenchymal stem cell therapies for liver cirrhosis, which is now already clinically available. As described in the article, more than 50 clinical trials related to liver diseases have already been conducted or are underway.

Leaders in the field of cardiac regenerative medicine are Drs. Fujita and Fukuda from Keio University who describe cell therapy using human iPS cell-derived cardiomyocytes for patients with severe heart failure. They are serious researchers on regeneration in cardiac medicine, and their review article shows the feasibility of their method for curing the patients. The start-up, Heartseed, Inc., was established to move that research forward.

Here, I am indebted to the contributors to this special issue for sharing their time and expertise and look forward to seeing further research flow from their work by providing clinical applications for regenerative medicine therapies.

